# One-year emergency department visits for children < 18 years of age, associated factors and frequency of primary general practitioner or pediatrician visits before: a French observational study (2018–19)

**DOI:** 10.1186/s12875-024-02328-1

**Published:** 2024-03-13

**Authors:** Jeanne Pergeline, Thomas Lesuffleur, Jeanne Fresson, Annick Vilain, Antoine Rachas, Philippe Tuppin

**Affiliations:** 1 Direction de la Stratégie, des Etudes et des Statistiques, Caisse nationale de l’assurance maladie, 26-50, Avenue du Professeur André Lemierre, Paris, Cedex 20 75986 France; 2 Direction de la recherche, des études, de l’évaluation et des statistiques, 10 place des cinq martyrs du lycée Buffon, Paris, 75014 France

**Keywords:** Administrative claims, Children, Chronic diseases, Ambulatory healthcare use, Emergency service, High users, Hospitalisation

## Abstract

**Background:**

This study was designed to identify factors associated with at least one emergency department (ED) visit and those associated without consultation by a general practitioner or paediatrician (GPP) before ED visit. Levels of annual consumption of healthcare services as a function of the number of ED visit were reported.

**Methods:**

This retrospective study focused on children < 18 years of age living in mainland France and followed for one-year after their birth or birthday in 2018. Children were selected from the national health data system, which includes data on healthcare reimbursements, long-term chronic diseases (LTD) eligible for 100% reimbursement, and individual complementary universal insurance (CMUc) status granted to households with a low annual income. Adjusted odds ratios (OR) were estimated using multivariate logistic regression.

**Results:**

There were 13.211 million children included (94.2% of children; girls 48.8%). At least one annual ED visit was found for 24% (1: 16%, 2: 5%, 3 or more: 3%) and 14% of visits led to hospitalization. Factors significantly associated with at least one ED visit were being a girl (47.1%; OR = 0.92), age < 1 year (9.1%; OR = 2.85), CMUc (22.7%, OR = 1.45), an ED in the commune of residence (33.3%, OR = 1.15), type 1 diabetes (0.25%; OR = 2.4), epilepsy (0.28%; OR = 2.1), and asthma (0.39%; OR = 2.0). At least one annual short stay hospitalisation (SSH) was found for 8.8% children of which 3.4% after an ED visit. A GPP visit the three days before or the day of the ED visit was found for 19% of children (< 1 year: 29%, 14–17 years: 13%). It was 30% when the ED was followed by SSH and 17% when not. Significant factors associated with the absence of a GPP visit were being a girl (OR = 0.9), age (1 year OR = 1.4, 14–17 years OR = 3.5), presence of an ED in the commune of residence (OR = 1.12), epilepsy LTD (OR = 1.1).

**Conclusion:**

The low level of visits to GPP prior to a visit to the ED and the associated factors are the elements to be taken into account for appropriate policies to limit ED overcrowding. The same applies to factors associated with a visit to the ED, in order to limit daily variations.

## Background

Children's visits to emergency departments (ED) are a major concern, as they can lead to overcrowding, pressure to transfer patients and variations in the quality of care. Studies have been carried out to assess the different socio-demographic characteristics, healthcare systems and policies between countries, as for adults. Some have looked at trends and the proportion of children among all ED visits, as well as independent factors associated with occasional or heavy use of ED. For example, in the US, children under 21 accounted for 25% of all ED visits in 2014 [1]. In Korea, children under 19 accounted for 31% of ED visits in 2010 [2]. Other studies have reported an increase in ED visits by children under 15 in England (2007–17: 1.5%/year) and Ontario (0–4 years: 43.2% to 55.4% from 2008 to 2018) [3, 4]. In Lombardy (Italy), attendance revealed that 27% of young people had visited the ED at least once in 2012, and 79% of them had done so for non-urgent reasons [5]. The most frequent diagnoses were trauma (26%) and respiratory tract infections (22%). In the United States, the most frequent pathologies for ED visits by children aged < 21 years were also trauma (26%), ear, nose and throat and dental or oral disorders (22%), gastrointestinal diseases (17%) and respiratory diseases (16%) [1]. Data for France are scarce, but children under 15 accounted for 27% of ED visits on a given day in 2013. Trauma accounted for 46% of visits and gastroenterology for 12% in 2013 [6, 7].

Many factors have been shown to be associated with occasional or high-frequency ED visits by children, such as sex, age, level of health insurance coverage and type, social deprivation of the children’s family or neighbourhood, vulnerability and complex chronic conditions [8–12]. Studies have also been performed to increase the information available on emergency ED use or not, such as specific qualitative factors like more frequent evening visits, language barrier and inappropriately use [13–15]. Moreover, studies also focused on ED frequent visitors and related factors [16–19]. Besides, other studies and reviews reported the reasons for inappropriately use of ED including the role of primary care before visit, less frequently studied [19–21].

Thus, the purposes of this nationwide observational study on 13.2 million children under the age of 18 years included in 2018 and followed for one-year were: 1) to evaluate the frequency of ED visits and identify factors associated with an ED visit; 2) those for the absence of a primary care consultation with a GPP before an ED visit; 3) to compare annual levels of various others healthcare use according to frequency of ED visit.

## Methods

### Population

**Fig. 1:**
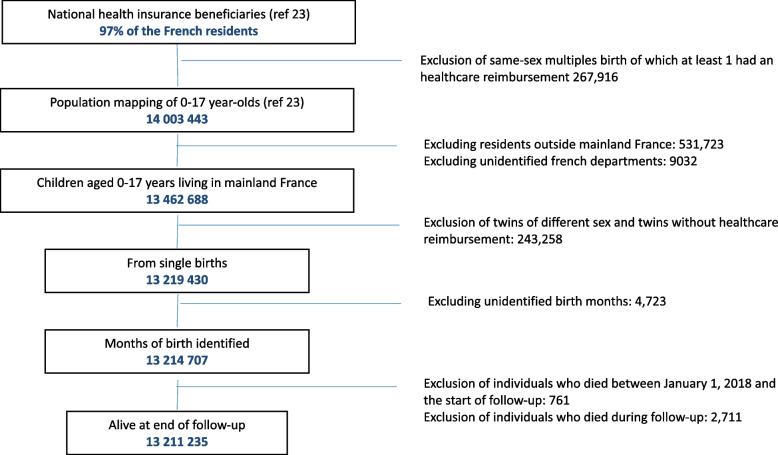
Flow chart to the selected children < 18 years-old in 2018

In mainland France (population of 64.9 million), there were around 14 million children < 18 years of age on the first of January 2019 according to the INSEE [Institut national de la statistique et des études économiques, National Institute for Statistics and Economic Studies]. This retrospective observational study included children < 18 years of age in 2018 with at least one healthcare expenditures reimbursed by the national health insurance (mapping population) and exclusion criteria in the chart flow (Fig. 1) [22]. They were followed up for one year between 2018.

### Data source

The national health data system (SNDS) includes data on individual sociodemographic characteristics, reimbursed primary care prescriptions, examinations and procedures, and public or private hospitalisations for almost the entire population with universal health insurance coverage [23]. The SNDS collects comprehensive anonymous data concerning all reimbursed prescriptions, examinations, and procedures performed in the outpatient setting, and hospitalisations. Information about the beneficiaries themselves (date of birth, sex, commune of residence, deprivation index etc.) are also available. This database does not record primary medical diagnoses or the results of clinical examinations and investigations. To obtain a long-term chronic disease status, a request by the patient’s (GP) is necessary and accepted by the national health insurance. It guarantees 100% reimbursement for all healthcare expenditures related to a long term disease (LTD) for at least five years and can be renewed according to disease evolution and clinical status. Diagnoses are made and validated after medical examinations, and, in some cases, hospitalisation or chronicization during the follow-up by the patient’s GP using national recommendations for diagnosis, treatment, and follow-up. The list of LTD is published and updated by decree after expertise from the HAS (Haute Autorité de Santé, French National Authority for Health) [24].

Through a pseudonymised identification number, this information is all linked, via the national hospital discharge database, to information concerning public and private hospital stays: ED visit, admission to short-stay hospitalisation (SSH), psychiatric hospitals or rehabilitation facilities, or home hospitalisation. Hospital diagnoses for SSH and LTD diagnoses are coded according to the International Classification of Diseases 10th revision (ICD-10).

A social deprivation index (FDep) has been constructed at the municipality scale (smallest administrative unit, 36,000 units in mainland France) according to four factors resulting from data published by the INSEE: average household income, the percentage of secondary school graduates (those who finished secondary school in France who passed the Baccalaureate exam) among inhabitants aged 15 and over, the percentage of blue-collar workers in the active population, and unemployment levels. It was computed in 2015 and is divided into five quintiles (1: most deprived, 5: least deprived) [10, 25]. The index could not be calculated for certain rural municipalities of metropolitan France. Children living in these municipalities were excluded from the specific analyses. Information on the type of commune of residence (urban or rural) is also present in the SNDS. Data concerning the presence of an ED in the municipality on December 31, 2019, were available from the Ministry of Health (DRESS) open data.

Another sociodeprivation marker is being the beneficiary, or not, of universal complementary health coverage (CMUc: couverture maladie universelle complémentaire), which is granted for one year (renewable) based on annual income to those who have had a stable and regular residence in France for over three months. The household can include the applicant, his/her spouse, partner, and their children. In 2018, the annual income limit was 8,810 euros for a single person and increased according to the number of people in the household. This limit is below the poverty threshold defined as 50% of the median annual income or 10,620 euros in France in 2018. The CMUc enables beneficiaries to access treatment without advancing costs with a reimbursement of 100% and without exceeding reimbursed costs [10, 26]. Children were classified as being a CMUc beneficiary if they had at least one specific outpatient reimbursement covered by the CMUc in 2018 or 2019.

### Outcomes

The first outcome was at least one ED visit during the year of follow-up and associated factors. The second outcome was to explore the proportion of children with an ambulatory GPP consultation within the three days before and the day of the ED visit and also associated factors. Individual characteristics and annual consumption levels of various healthcare use according to number of ED visit were reported.

### Analyses

We first analyzed the children’s sociodemographic characteristics, type of municipality of residence, and the presence of an ED in the municipality of residence according to the number of ED visits (none, 1, 2, ≥ 3 during the year) Then, we analyzed those children who had at least one visit to a healthcare service during the year of follow-up according to the number of ED visits to explore the level of their use according to the frequency of ED visits. To assess the intensity of healthcare use during the year, the median and interquartile range (IQR) of the number of visits were calculated. Data on the LTD diagnosis are expressed as the proportion of at least one LTD. The 10 most frequent LTD among the entire population were also studied and others grouped together.

Logistic regression was used to estimate odds ratios (OR) of the associations between at least one annual ED visit and the factors listed above. The FDep was not considered in the full analysis due to collinearity with CMUc, and this index was adjusted only for age and sex. This was also true for those with at least one of all LTD, in this case the 10 most frequent LTD diagnoses or those of interest were included individually and the others grouped in a specific category of other LTD diagnoses. For each outcome, the odds ratio (OR) and 95% confidence interval (95%CI) were calculated and then adjusted by sex and age and finally the full model was generated using factors selected from literature and then their presence or not in the SNDS. The same method was used to determine the presence of at least one GPP visit the three days before and the day of the ED visit. Given the almost exhaustive nature of the population and the large sample size, we report crude and adjusted OR without 95% confidence intervals [27].

SAS software (version 7.13, SAS Institute Inc, Cary, NC, USA) and R software (4.1.2) were used for the statistical analysis.

## Results

### Population characteristics and healthcare use

The under-18 mapping population, around 14 million of children with at less one reimbursement, underwent several exclusion stages (Fig. 1). Their main purpose was to eliminate children of the same sex from multiple pregnancies, indistinguishable in the SNDS, and for the sake of homogeneity, those of different sexes. Children not residing in mainland France were also excluded. Then, children with coding problems and those who died during the one-year follow-up were not included. Finally, 13.211 million children were included (94.2%) Overall, this study concerned singleton children living in mainland France with national health insurance coverage who had at least one healthcare reimbursement from health insurance in 2018.

Their median age was 9 years [IQR = 4–13] and decreased with the number of ED visits (4 years for ≥ 3 ED) (48.8% girls) (Table 1). CMUc was identified for 17.5% of children and their proportion was similar (approximately 20%) for each deprivation quintile. Close to 22% lived in a rural municipality and 29% in a municipality with an ED. At least one LTD was noted for 4.0% of the children (boys 4.6%, girls 3.3%). Six of the 10 most frequent LTD were classified as “mental and behavioural disorders”, the most frequent being “pervasive developmental disorders” (0.53%). Asthma (0.24%) was the most frequent somatic LTD, followed by epilepsy (0.17%) and type 1 diabetes (0.15%).

**Table 1:** Sociodemographic characteristics and the most frequent long-term diseases diagnoses of children < 18 years of age in 2018 and followed for one year after their birthday or birth according to the number of emergency department visits

	**Total**	**One-year emergency department visit**					**Overall age**
		**No**	**At least one**	**1**	**2**	**≥3**	**Median IQR**
N (million)	13.211	10.096	3.115	2.131	0.615	0.369	
% row	100.0	76.4	23.5	16.1	4.7	2.8	
N ED visits (million)	4,805	0	4,805	2,131	1,230	1,444	
% row			100.0	44.3	25.6	30.1	
	**%**	**%**	**%**	**%**	**%**	**%**	
**Age**							-
0 - < 1 year	5.1	3.9	9.1	7.1	10.9	17.7	-
1 - < 2	5.3	4.2	8.8	7.4	10.4	14.0	-
2 - < 5	16.4	15.2	20.3	19.6	21.5	21.8	-
5 - < 10	28.4	29.9	23.7	25.7	21.5	16.1	-
10 - < 14	22.7	23.8	18.9	20.1	17.6	14.4	-
14 - < 18	22.1	23.0	19.2	20.1	18.2	16.0	-
Median [IQR]	9 [4–13]	9 [5–13]	7 [2–12]	8 [3–12]	6 [2–12]	4 [1–11]	-
**Gender**							
Girls	48.8	49.4	47.1	46.8	47.1	48.8	9 [4–13]
Boys	51.2	50.6	52.9	53.2	52.9	51.2	9 [4–13]
**CMUc**	17.5	16.0	22.7	20.6	24.7	31.4	8 [4–12]
No CMUc	82.4	83.9	77.1	79.2	75.2	68.5	9 [4–13]
*Missing*	0.1	0.1	0.2	0.2	0.1	0.1	
**FDep deprivation index (quintile)**							
1 (less deprived)	19.5	20.4	16.6	17.3	15.8	14.2	9 [4–13]
2	20.0	20.4	18.7	19.2	18.1	16.9	9 [4–13]
3	19.6	19.5	20.1	20.1	20.2	20.3	9 [4–13]
4	19.5	19.2	20.5	20.4	20.7	20.7	9 [4–13]
5 (most deprived)	20.1	19.3	23.0	22	24.2	27.0	9 [4–13]
*Missing*	1.1	1.1	1.1	1.1	1.1	1.0	
**Municipality of residence**							
Urban	77.5	77.0	79.4	78.5	80.2	82.9	8 [4–13]
Rural	21.8	22.4	20.0	20.9	19.2	16.6	9 [5–13]
*Missing*	0.6	0.7	0.6	0.6	0.6	0.5	
**ED in the municipality residence**							
Yes	29.5	28.3	33.3	31.9	34.8	39.0	8 [4–13]
No	69.5	70.6	65.8	67.2	64.3	60.2	9 [4–13]
*Missing*	1	1	1	1	0.9	0.8	
**Total**							
At least one LTD	4.0	3.5	5.3	4.6	5.8	8.8	10 [6–14]
No LTD	96.0	96.5	94.7	95.4	94.2	91.2	8 [4–13]
**Boys**							
At least one LTD	4.6	4.2	5.8	5.2	6.3	9.1	10 [6–14]
No LTD	95.4	95.8	94.2	94.8	93.7	90.9	8 [4–13]
**Girls**							
At least one LTD	3.3	2.9	4.8	4.0	5.3	8.4	11 [7–14]
No LTD	96.7	97.1	95.2	96.0	94.7	91.6	9 [4–13]
**10 most frequent LTD (ICD-10):**							
Pervasive developmental disorders	0.53	0.52	0.56	0.53	0.58	0.68	10 [7–13]
Asthma	0.24	0.19	0.39	0.30	0.44	0.80	9 [5–13]
Specific developmental disorders of speech and language	0.17	0.17	0.19	0.18	0.19	0.21	10 [7–13]
Epilepsy	0.17	0.13	0.28	0.21	0.32	0.62	11 [7–14]
Unspecified mental retardation	0.16	0.15	0.18	0.17	0.19	0.25	11 [7–14]
Type 1 diabetes mellitus	0.15	0.12	0.25	0.23	0.28	0.34	12 [9–15]
Scoliosis	0.15	0.16	0.13	0.13	0.12	0.13	15 [13–16]
Specific developmental disorders of scholastic skills	0.12	0.12	0.13	0.12	0.13	0.14	11 [9–14]
Mixed disorders of conduct and emotions	0.10	0.09	0.13	0.11	0.13	0.20	12 [9–14]
Mixed specific developmental disorders	0.09	0.08	0.10	0.1	0.12	0.13	10 [7–13]

*ED* emergency department, *IQR* Interquartile range, *LTD* long term disease, *ICD-10* the International Classification of Diseases 10th Revision, *CMUc* complementary universal health insurance coverage

**Fig. 2:**
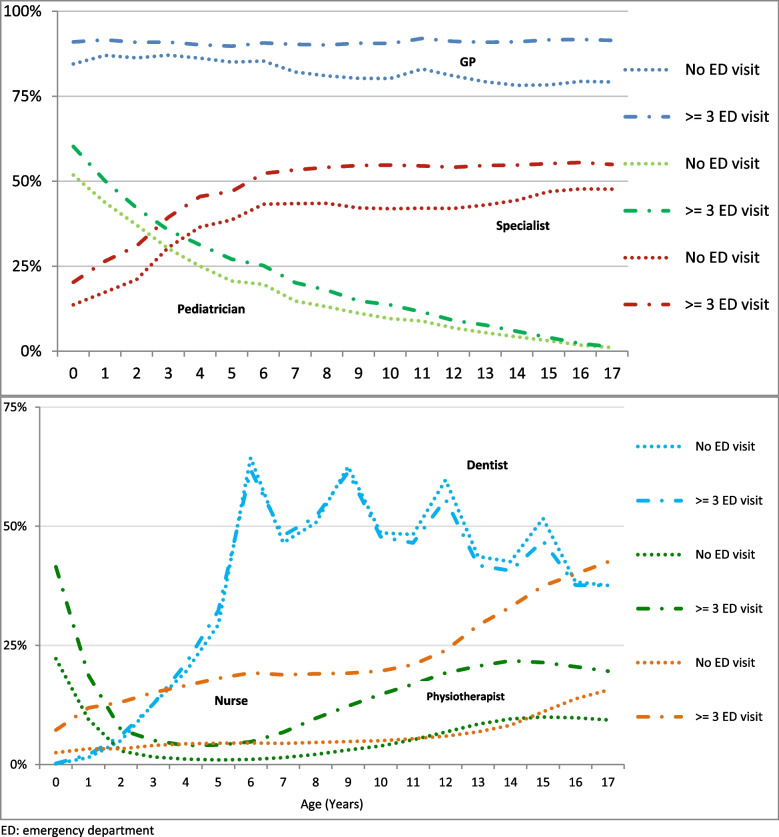
At least one annual visit to a healthcare professional by children < 18 years of age in 2018 and followed for one year after their birthday or birth according to age and three ED visits or more vs none

At least one annual GPP visit was found for 88% of children (median 3, IQR = 2–6): GP only (83.6%), paediatrician only (17%), other specialist (39.5%), nurse (8.1%), and physiotherapist (7%) (Table 2). Age varied as a function of the type of visit: mainly a lower age for visits to a paediatrician and a steady increase for visits to a specialist up to the age of six years (Fig. 2). At least one annual stay in SSH was found for 8.8% children (median 1, IQR = 1–1), of which 3.4% after an ED visit. In addition, there was at least one annual stay for 0.31% in a psychiatric hospital and 0.23% had hospitalisation at home.

**Table 2:** Use of healthcare services over one year by subjects < 18 years of age in 2018 and followed for one year after their birth or birthday according to the number of emergency department visits

		**One-year emergency department visit**				
	**Total**	**No**	**At least one**	**1**	**2**	** ≥ 3**
N children (million)	13,211	10,096	3,115	2,131	0,615	0,369
	%	%	%	%	%	%
**Primary care consultation**^**a**^						
**GPP (%)**^**a**^	88.0	86.7	92.3	91.4	93.6	95.4
Median [IQR]	3 [2–6]	3 [2–5]	4 [2–8]	4 [2–7]	5 [3–8]	6 [3–11]
**GP (%)**^**a**^	83.6	82.2	88.0	87.1	89.3	91.0
Median [IQR]	3 [2–5]	3 [1–5]	4 [2–6]	3 [2–6]	4 [2–7]	5 [3–9]
**Paediatrician (%)**^**a**^	17.3	15.6	22.6	20.5	24.8	31.3
Median [IQR]	2 [1–4]	2 [1–4]	3 [1–6]	2 [1–5]	3 [1–6]	4 [2–8]
**Nurse (%)**^**a**^	8.1	6.4	13.6	12.2	15.5	18.7
Median [IQR]	1 [1, 2]	1 [1, 2]	1 [1–4]	1 [1–3]	1 [1–5]	2 [1–6]
**Physiotherapist (%)**^**a**^	7.1	5.7	11.4	9.7	13.1	18.3
Median [IQR]	8 [4–15]	8 [4–15]	8 [4–15]	8 [4–15]	7 [4–14]	7 [4–15]
**Dentist (%)**^**a**^	37.3	39.0	31.8	33.7	29.7	24.4
Median [IQR]	1 [1, 2]	1 [1, 2]	1 [1, 2]	1 [1, 2]	1 [1, 2]	1 [1, 2]
**Specialists (except paediatrician (%)**^**a**^	39.5	39.2	40.3	40.2	40.6	40.5
Median [IQR]	1 [1, 2]	1 [1, 2]	1 [1, 2]	1 [1, 2]	1 [1, 2]	1 [1, 2]
**Ophthalmologist**^**a**^	24.7	25.3	23.0	23.6	22.4	20.4
**Dermatologist**^**a**^	7.1	7.3	6.5	6.6	6.3	5.8
**ENT**^**a**^	7.0	6.5	8.7	8.2	9.4	10.8
**Surgeon**^**a**^	4.2	3.5	6.5	6.0	7.4	8.1
**Cardiologist**^**a**^	1.2	1.1	1.6	1.5	1.7	2.2
**Pneumologist**^**a**^	1.2	1.1	1.4	1.3	1.5	1.8
**Psychiatrist**^**a**^	1.0	1.0	1.2	1.1	1.2	1.4
**Outpatient consultation**^**a**^						
GP or paediatrician (%)^a^	8.5	5.0	19.9	17.4	22.8	29.1
GP (%)^a^	5.6	2.9	14.1	13.1	15.9	17.0
Paediatrician (%)^a^	3.1	2.2	6.4	4.73	7.6	13.7
**Hospitalisation**^**a**^						
**SSH**	8.8	4.84	21.5	16.2	26.93	42.51
Median [IQR]	1 [1–1]	1 [1–1]	1 [1–1]	1 [1–1]	1 [1–1]	1 [1, 2]
**SSH after ED visit**	3.4		14.3	9.5	19.1	34.0
**Paediatric Intensive care**	0.10	0.03	0.30	0.16	0.39	0.95
**Neonatology**	0.60	0.35	1.30	0.88	1.58	3.26
**Neonatology with intensive care**	0.20	0.12	0.49	0.31	0.60	1.37
**Psychiatric hospitalisation**	0.3	0.22	0.59	0.41	0.69	1.49
**Hospitalisation at home**	0.2	0.19	0.35	0.07	0.14	0.63
**Rehabilitation care**	0.0	0.02	0.11	0.29	0.41	0.32

*GPP* general practitioner or paediatrician, *GP* general practitioner, *IQR* Interquartile range, *ED* emergency department, *SSH* short stay hospital

^a^At least one during the follow-up

### ED visits

Among all children, 24% had at least one annual ED visit: 1 (16.1%), 2 (4.7%), or 3 or more (2.8%). Among ED visits, 44.4% corresponded to one ED visit during the year, two to 25.6%, and 30.0% to 3 ED visits or more.

Children, who had at least one ED visit were younger (median 7, IQR: 2–12) than those who did not have an ED visit (median 9, IQR: 5–13) (Table 1). They also more often had CMUc (23% vs 16%), a residence in the highest deprivation quintile (23% vs 19%) and in an urban area (79% vs 77%), an ED located in their municipality (33% vs 28%), and at least one LTD (5.3% vs 3.5%): mainly asthma, epilepsy, diabetes, or a psychiatric condition. They had also more frequently at least one primary care professional visit during the year of follow-up (including ED visit): GPP (92.3% vs 86.7%), GP (92% vs 87%), pediatrician (23% vs 16%), or some other medical specialist: surgeon (6.5% vs 3.5%), ENT (9% vs 6.5%), nurse (24% vs 6%), or physiotherapist (11% vs 6%). They were also more often admitted for SSH (21% vs 5%) and other types of hospitalization, mainly psychiatric (0.6% vs 0.2%). These frequencies increased with the number of ED visits (Tables 1 and 2).

The full model comparing children who had at least one ED visit to those who did not showed significantly lower ED use for girls (OR = 0.92) and higher ED use before the age of five years (mainly for children < 1 year OR = 2.85) (Table 3). Other factors that significantly increased the likelihood of visiting an ED were having CMUc (OR = 1.45), an ED in the commune of residence (OR 1.15), and mainly for somatic LTD: asthma (OR = 2.0), epilepsy (OR = 2.1), diabetes mellitus (OR = 2.4).

**Table 3:** Sociodemographic characteristics and long-term disease status associated with at least one ED visit vs no visit among subjects < 18 years of age in 2018 and followed for one year after their birth or birthday

	**Odds ratio** **(95% CI)**	**Odds ratio adjusted by** **age and gender (95% CI)**	**Odds ratio adjusted** **Full model (95% CI)**
**Age (Years)**			
< 1	2.84 [2.82–2.85]		2.85 [2.84–2.87]
1	2.49 [2.47–2.50]		2.47 [2.46–2.49]
2–4	1.60 [1.59–1.60]		1.58 [1.57–1.59]
5–9	0.95 [0.94–0.95]		0.94 [0.94–0.94]
10–13	0.95 [0.95–0.95]		0.95 [0.94–0.95]
14–17	1		1
**Gender**			
Boys	1		1
Girls	0.91 [0.91–0.91]		0.92 [0.91–0.92]
**CMUc vs none**	1.55 [1.55–1.56]	1.52 [1.51–1.52]	1.45 [1.44–1.45]
**FDep deprivation index (quintile)**			
1 (less deprived)	1	1	
2	1.12 [1.12–1.13]	1.13 [1.12–1.13]	
3	1.27 [1.26–1.27]	1.28 [1.28–1.29]	
4	1.31 [1.30–1.31]	1.33 [1.32–1.33]	
5 (most deprived)	1.47 [1.46–1.48]	1.49 [1.48–1.49]	
**Commune type**			
Urban	1	1	1
Rural	0.87 [0.87–0.87]	0.89 [0.89–0.89]	0.99 [0.99–0.99]
**ED in the commune of residence vs no**	1.26 [1.26–1.27]	1.23 [1.23–1.24]	1.15 [1.15–1.15]
**At least one LTD vs no**	1.53 [1.52–1.54]	1.70 [1.69–1.72]	
**LTD**			
Pervasive developmental disorders	1.06 [1.04–1.08]	1.21 [1.19–1.23]	1.08 [1.06–1.10]
Asthma	2.06 [2.01–2.11]	2.23 [2.18–2.28]	2.04 [1.99–2.09]
Specific developmental disorders of speech and language	1.11 [1.08–1.14]	1.30 [1.27–1.34]	1.17 [1.13–1.20]
Epilepsy	2.16 [2.11–2.22]	2.45 [2.39–2.52]	2.13 [2.07–2.19]
Unspecified mental retardation	1.20 [1.16–1.24]	1.39 [1.35–1.43]	1.12 [1.09–1.16]
Type 1 diabetes mellitus	2.08 [2.02–2.14]	2.49 [2.42–2.56]	2.39 [2.32–2.46]
Scoliosis	0.80 [0.77–0.83]	1.00 [0.96–1.03]	0.97 [0.94–1.01]
Specific developmental disorders of scholastic skills	1.11 [1.07–1.15]	1.34 [1.30–1.39]	1.20 [1.16–1.24]
Mixed disorders of conduct and emotions	1.38 [1.33–1.43]	1.65 [1.59–1.72]	1.43 [1.38–1.49]
Mixed specific developmental disorders	1.26 [1.21–1.31]	1.45 [1.39–1.51]	1.26 [1.21–1.31]
Congenital malformations of cardiac septa	1.60 [1.54–1.67]	1.55 [1.49–1.61]	1.32 [1.27–1.38]
At least one other LTD	1.68 [1.67–1.69]	1.81 [1.80–1.83]	1.72 [1.70–1.73]

*95% CI* 95% confidence interval, *CMUc* complementary universal health insurance coverage, *LTD* long term disease, *ED* emergency department

### GP or paediatrician visit before an ED visit

Among all children who had an ED visit, 8.8% had at least one GPP visit in the same day and 10% the 3 days before. For children < 1 year of age, the respective values were 11% and 17% and for those 14–17 years of age, 6.8% and 6.2% (Table 4). A GPP visit the same day was more frequent for ED visits following by SSH (15% vs 8%) and decreasing with age. There was no marked difference in the frequency of GPP visits and ED visit according to CMUc, FDep quintile, having at least one LTD, or having an ED in the commune.

**Table 4:** Percentage of children with an ambulatory visit with a general practitioner or paediatrician (GPP) for the month before an emergency department visit among children < 18 years of age in 2018 followed for one year after their birth or birthday among risk factors

	**Total**	**Age (years)**						**CMUc**		**FDep**					**LTD**		**ED in the commune of residence**		**ED visit**		
		** < 1**	**1**	**2–4**	**5–9**	**10–13**	**14–17**	**Yes**	**No**	**1**	**2**	**3**	**4**	**5**	**Yes**	**No**	**Yes**	**No**	**First**	**Second**	**Third or more**
**All ED visits N (millions)**	4,805	0,563	0,494	0,992	1,029	0,843	0,884	1,222	3,577	0,762	0,872	0,969	0,986	1,165	0,314	4,491	1,684	3,077	2,131	1,230	1,444
***% row***	100.0	11.7	10.3	20.6	21.4	17.5	18.4	25.4	74.4	15.9	18.1	20.2	20.5	24.2	6.5	93.5	35.0	64.0	44.4	25.6	30.0
	%	%	%	%	%	%	%	%	%	%	%	%	%	%	%	%	%	%	%	%	%
**Same day (day 0)**	8.8	11.3	10.1	9.1	9.0	7.9	6.8	8.1	9.1	9.4	10.0	9.2	8.5	7.4	8.8	11.3	10.1	9.1	9.0	7.9	6.8
1 day before	4.3	6.9	6.6	4.9	3.8	3.0	2.8	4.5	4.3	4.7	4.7	4.4	4.2	4.0	4.3	6.9	6.6	4.9	3.8	3.0	2.8
2–3 days	5.5	10.3	8.7	6.3	4.3	3.4	3.4	5.7	5.5	5.8	5.8	5.5	5.3	5.4	5.5	10.3	8.7	6.3	4.3	3.4	3.4
**ED followed by SSH N**	0,527	0,127	0,062	0,092	0,082	0,067	0,096	0,141	0,386	0,082	0,095	0,105	0,110	0,129	0,070	0,457	0,178	0,345	0,202	0,135	0,191
***% row***	100.0	24 .1	11 .8	17 .5	15 .6	12 .7	18 .2	26 .8	73 .2	15 .6	18 .0	19 .9	20 .9	24 .5	13 .3	86 .7	33 .8	65 .5	38 .3	25 .6	36 .2
	%	%	%	%	%	%	%	%	%	%	%	%	%	%	%	%	%	%	%	%	%
**Same day (day 0)**	14.7	16.2	15.9	15.6	16.4	14.2	10.1	12.1	15.7	15.4	16.2	14.9	14.7	13.0	14.7	16.2	15.9	15.6	16.4	14.2	10.1
1 day before	7.4	8.6	10.4	8.6	6.9	5.5	4.5	6.6	7.7	7.7	7.8	7.6	7.3	6.8	7.4	8.6	10.4	8.6	6.9	5.5	4.5
2–3 days	8.0	11.6	11.1	8.8	6.2	4.8	4.6	7.4	8.3	8.0	8.3	8.2	7.8	8.0	8.0	11.6	11.1	8.8	6.2	4.8	4.6
**ED without SSH N**	4,288	0,44	0,43	0,90	0,95	0,78	0,79	1,08	3,19	0,68	0,78	0,86	0,88	1,04	0,24	4,03	1,51	2,73	1,93	1,10	1,25
***% row***	10,2	10,1	21,0	22,1	18,1	18,4	25,3	74,6	15,9	18,1	20,2	20,5	24,2	5,7	94,3	35,2	63,9	45,1	25,6	29,3	10,2
	%	%	%	%	%	%	%	%	%	%	%	%	%	%	%	%	%	%	%	%	%
**Same day (day 0)**	8,1	10,0	9,3	8,4	8,4	7,4	6,4	7,6	8,3	8,7	9,3	8,5	7,7	6,8	7,4	8,1	7,5	8,4	8,6	8,1	7,3
1 day before	4,0	6,5	6,0	4,5	3,5	2,8	2,6	4,2	3,9	4,3	4,3	4,0	3,8	3,6	3,9	4,0	3,8	4,1	3,6	4,1	4,5
2–3 days	5,2	10,0	8,3	6,1	4,1	3,2	3,3	5,5	5,2	5,5	5,5	5,2	5,0	5,1	5,1	5,2	5,1	5,3	4,4	5,4	6,5

*CMUc* complementary universal health insurance coverage, *ED* emergency department, *LTD* long term disease FDep deprivation index (quintile)

The absence of at least one GPP visit the three days before or the day of the ED visit (Table 5) increased with the social deprivation quintile (adjusted for age and sex only). The use of all other factors showed that the absence of at least one GPP visit increased with age and the presence of an ED in the commune of residence. However, the OR was < 1 for girls and most of the LTD except epilepsy.

**Table 5:** Association of sociodemographic characteristics and long-term disease with the absence of a GPP visit in the three days before or the day of an ED visit among subjects < 18 years of age in 2018 and followed one year after their birth or birthday

	**Odds ratio** **(95% CI)**	**Odds ratio adjusted** **age and gender (95% CI)**	**Odds ratio adjusted** **Full model (95% CI)**
**Age (Years)**			
< 1	1		1
1	1.41 [1.39–1.42]		1.41 [1.40–1.42]
2–4	1.96 [1.94–1.97]		1.96 [1.95–1.98]
5–9	2.65 [2.63–2.68]		2.68 [2.65–2.70]
10–13	3.35 [3.31–3.38]		3.40 [3.36–3.43]
14–17	3.40 [3.36–3.43]		3.46 [3.43–3.50]
**Gender**			
Boys	1		1
Girls	0.94 [0.93–0.94]		0.91 [0.90–0.91]
**CMUc vs no**	0.97 [0.96–0.98]	1.0 [0.99–1.01]	0.97 [0.97–0.98]
**FDep deprivation index (quintile)**			
1 (less deprived)	1	1	
2	1.00 [0.99–1.01]	0.98 [0.97–0.99]	
3	1.06 [1.05–1.07]	1.04 [1.03–1.05]	
4	1.11 [1.10–1.12]	1.08 [1.07–1.09]	
5 (most deprived)	1.15 [1.13–1.16]	1.13 [1.12–1.14]	
**Commune type**			
Urban	1	1	1
Rural	1.04 [1.03–1.04]	0.97 [0.96–0.98]	1.01 [1.00–1.02]
**ED in the commune of residence vs no**	1.07 [1.06–1.08]	1.11 [1.1–1.12]	1.12 [1.11–1.13]
**At least one LTD vs no**	1.06 [1.05–1.07]	0.89 [0.88–0.90]	
Pervasive developmental disorders	1.21 [1.16–1.26]	0.91 [0.88–0.95]	0.92 [0.88–0.95]
Asthma	0.79 [0.76–0.82]	0.72 [0.69–0.74]	0.73 [0.70–0.75]
Specific developmental disorders of speech and language	1.41 [1.31–1.51]	1.04 [0.96–1.12]	1.05 [0.98–1.14]
Epilepsy	1.25 [1.19–1.31]	1.06 [1.01–1.12]	1.09 [1.04–1.15]
Unspecified mental retardation	1.09 [1.02–1.16]	0.84 [0.79–0.90]	0.85 [0.80–0.91]
Type 1 diabetes mellitus	0.93 [0.89–0.98]	0.67 [0.63–0.70]	0.67 [0.64–0.71]
Scoliosis	1.23 [1.13–1.34]	0.83 [0.76–0.90]	0.84 [0.77–0.92]
Specific developmental disorders of scholastic skills	1.41 [1.29–1.55]	0.97 [0.89–1.07]	1.00 [0.91–1.09]
Mixed disorders of conduct and emotions	1.54 [1.41–1.68]	1.03 [0.94–1.12]	1.03 [0.94–1.12]
Mixed specific developmental disorders	1.20 [1.10–1.31]	0.93 [0.85–1.02]	0.93 [0.85–1.02]
Congenital malformations of cardiac septa	0.83 [0.77–0.89]	0.89 [0.83–0.96]	0.92 [0.86–0.99]
At least one other LTD	1.05 [1.03–1.06]	0.92 [0.91–0.93]	0.93 [0.92–0.94]

*95% CI* 95% confidence interval, *CMUC* complementary universal health insurance coverage, *LTD* long term disease, *ED* emergency department

## Discussion

This large quasi-exhaustive observational study in mainland France on 13.211 million children aged < 18 years with a one-year follow-up (2018–2019) found at least one annual ED visit for approximately 24% of all children, of which 14% were followed by a SSH. In multivariate analysis, at least one ED visit was less frequent for girls and more frequent for the youngest and those with CMUc, an ED in the commune of residence, and an LTD for asthma, epilepsy, type 1 diabetes, or certain mental conditions. Overall, the frequency of a least one primary GPP visits the three days before or the same day of the ED visit was 19%. If the ED visit was followed or not by an SSH admission it was respectively 30% and 17% (15% and 8% the day of ED visit). In multivariate analysis, factors associated with the absence of a GPP consultation the three days before or the day of the ED visit were increasing age of the child, mainly at adolescence, being a boy, having an ED in the commune of residence, and the presence of a LTD (asthma, diabetes, etc.), but not for some, such as epilepsy, for which the child was probably directly transferred to an ED.

There are differences in the frequency of at least one annual ED visit by children between countries, particularly for high users, who account for a relatively large share of ED visits given their relatively low number. In our study, 24% of children < 18 years-old had at least one annual ED visit: 16% only one, 6.5% at least two, and 3% three or more, totalling 44%, 26%, and 30% of the ED visits, respectively. In Northwest London, among 0 to 15 year olds in 2019, the ED visit rate was similar to our study (< 18 years: 24%) [3]. This was also the case for an Italian region in 2012 with also 24% [5]. In the USA, between 2010 and 2014, only 12% of children (< 17 years) had visited an ED at least once during the year (18% for those < 3 years of age). On the contrary, the proportion of children with two visits or more was higher in the USA than in our study (21% vs 10%) [28]. In Montreal in 2014, 4.7% of children had five or more ED visits, accounting for 17% of all visits [16]. In California, 2.3% had more than five visits, accounting for 9.3% of all visits [17] and in the Netherlands, 5% of children had four or more visits, accounting for 21% of all visits [18]. In England, close to 10% of children visited an ED four or more times in a year [29]. In Ontario (0–4 years: 43.2% to 55.4% from 2008 to 2018) [4]. Increase and excessive repetition of ED visits generates additional excess costs as inappropriate ED visits, which could be limited by actions on the factors associated with repeated ED visits justified or not [18, 30]. Our study identifies factors that are more or less frequently reported associated with one ED visit or more such as younger age, male sex, low social status in the household or the area where the home is located, the existence of certain chronic pathologies, proximity to an ED [4, 5, 7, 10–12, 31].

Children with LTD in our study maybe be considered as similar to medically complex conditions reported in the USA: 0.67% to 11.4% in the population, depending on the definition, and higher for the most deprived children [31–33]. A study reported at least an annual ED visit around 20% [33]. By definition, LTD require regular, costly, long-term care, and may be potentially life-threatening or disabling. However, the prevalence of LTD of 4% reported here may be considered as relatively low relative to the prevalence reported for children with complex conditions to whom they may be compared. Nevertheless, certain children may not yet have been diagnosed or may have had low-intensity symptoms, with little or no use of healthcare services at the onset of their disease.

Moreover, another study on the same population reported that LTD were more frequent among children living in the context of deprivation (6% for those with CMUc vs 4% without) [10]. In the present study, at least one LTD was noted for 3.5% of children who did not have an ED visit, 5.3% for those who had at least one and 9% for those who had three or more. For some LTD, we can suppose that parents are well educated about the signs of chronic disease decompensation but they are not always with the child (school…). For the same population, another analysis pointed that among children with one hospitalisation during the study year, 56% had an ED visit, 42% if the hospitalisation was less than one night, 78% if the hospitalisation was for one night or more and 87% if it was a readmission > 1 night before 30 days [34].

Our one-year study did not concern adults and it does not allow comparisons. Detailed information on access to ED as well as examinations and procedures carried out during stay and diagnosis usually are lacking to analyse the results. However, a French nationwide survey of 52,000 people on a given day in june 2013 was performed (27% of individuals were under 15 and 6% under two) [6, 7]. A detailed questionnaire was filled by each patient or family and caregivers, including most of the lacking information cited above. This study reports high daily crude rate of ED visit for 1,000 inhabitants for extreme ages. It was 2/1,000 habitants for children < 1 year followed by a slow decrease to 1.1/1,000 at 10–14 years and then by an increase only after 75 years (1/1,000). These crude rates may therefore lead us to believe that children had more frequent ED visits than adults, as opposed to SSH after ED visit hospitalisation (10% vs 17%). This suggest that ED visits not followed by SSH are inappropriate.

In the 2013 study, rates are similar between children > 6 months and adults after adjusting on diagnosis distribution and hospitalisation department speciality [6, 7]. Diseases and their proportions are intrinsically different between children and adults in terms of severity, distribution of diagnostic with high hospitalisation rates as cardiovascular diseases for example, clinical presentation or sufficient treatment in ED not requiring hospitalisation as for some traumatology cases. The French 2013 study reported ED diagnoses by chapter. Their proportion varied according to age, with trauma being more frequent in children (46% vs 35%) while cardiological and neurological problems were more frequent among adults (4% vs 16%) than in children. The need for hospitalisation is therefore different, but care and management of minor trauma in ED can be considered as an emergency. The inappropriate nature of ED is therefore difficult to establish and frequency of exams during the ED visit were reported. During the ED stay in 2013, children and adults had similar percentage of conventional imaging (35%), less biological analyses (14% vs 37%) and more at least one non-clinical exams (50% vs 34%) and diagnostic procedures. For children, 6% arrived via emergency transport, and 15% of 15–74 year-olds. Adults arrival time was: 8am-5pm: 55%, 5pm-9pm: 23%, 9pm-12pm: 12% and for children it was quite similar except a pic at the end of the day (50%, 32%, 12%). This has also been reported, with factors linked to families' choice of emergency departments [11–19].

Studies on the factors associated with ED visits and limiting non-necessary ED visits or other preventable visits have been conducted in the search for individual or organisational risk factors associated with ED visits, SSHs, and their frequent use to reduce the volume and costs [35–38]. Several have reported an association between non-urgent ED visits and the sociodemographic characteristics of the child and family, limited access to primary care, reassurance and convenience of this mode of care available during holidays and weekends, and long-term conditions, which may be more often followed by hospital admission, as in our study. In the USA, 75% of ED visits occurred during the night and weekends in 2012, when less ambulatory care is available [35]. We found a relatively low GPP visit rate three days before an ED visit, which rose a little on the day of the visit. The rate of ambulatory visits on the same day may be slightly overestimated, because some GPP visits likely occurred after the ED visit, although doctors’ offices are more often closed at the end of the day and on weekends.

Studies were developed investigate the methods implemented to reduce inappropriate. One telephone triage was the single best-evaluated intervention for accident and emergency department attendances. For all other interventions considered in this review (walk-in centres, minor injuries units, and out-of-hours general practice), the effects on A&E attendance, patient outcomes, and cost were inconclusive [35]. Studies were also implemented to understand how the uptake of an extended primary care service in the evenings and weekend varied by day of week and over time [36]. A second similar study found that extending primary care to GP was associated with a reduction in emergency department visits in the first 12 months [37]. To expend policies, data on health professional density and volume of ED visits must be investigate. This was the case for the same study population [38]. Thus, the density of healthcare professionals in France varied between quintiles of the FDep: it has been shown to be higher for nurses for the highest deprivation quintiles, the same for GPs, and lower for other healthcare professionals, mainly psychiatrists and pediatricians. An analysis between *French administrative geographical units (départements)* found, after age standardisation, that the frequency of GP or paediatrician visits inversely correlated with the frequency of ED visits not followed by a SSH (correlation coefficient *r* = -0.30, *p* = 0.003). The frequency of seeing another specialist was also inversely correlated with the frequency of visiting an ED not followed by a SSH (*r* = -0.25, *p*-value = 0.02).

As we have seen throughout the text, this type and theme of study requires a great deal of information, much of which is not available in information systems and is very time-consuming. Nevertheless, it is possible to provide information through medical prescriptions at various times before and mainly after the ED visit, transfers or readmissions, acts or procedures during the following month and consultations.

### Strengths and limitations

The main strength of this study was the use of the SNDS, which allowed us to include more than 13 million children of the mainland French population of this age listed by the INSEE and which exhaustively collects primary healthcare and hospital discharge information. Excluding, children from overseas department We observed a difference with the INSEE population, which increased with age, possibly due to the non-inclusion of children who had no reimbursements during the year of this study, therefore leading to a slight overestimation of healthcare consumption. Most of the individuals who did not have reimbursements during the year appear to be adolescents. In addition, younger children born alive but not discharged from the hospital during the year may have not been included in the study because they did not have an outpatient refund and consequently their CMUc status could not be determined. The social deprivation index does not necessarily prejudge the social disadvantage of each individual living in a municipality and it was not entirely independent of the CMUc status at the individual level, which was also more frequent in more deprived quintiles.

In addition, the rate may have been slightly underestimated for the youngest patients because some doctor’s visits may have occurred in maternity and child welfare centres (PMI: protection maternelle et infantile). These centres are public and carry out medical and social prevention, but not emergency care, for mothers and their children aged < 6 years, and no reimbursement is needed for free visits, approximately 2% under two years were not collected in the SNDS [22]. This study collected and studied data concerning all ED visits but we cannot estimate the proportion of ED visits that were avoided due to upstream medical visits or specific care and support.

## Conclusions

Our results for this and complementary studies offer insightful baseline information on variations in healthcare use by children. These data must also be considered with those of specific studies, such as at the regional level, to adapt policies and future research according to multiple characteristics, as well as plan and adapt the provision of care to the needs and access requirements at different levels to the different steps of the classic care path of children.

## Data Availability

All SNDS data are anonymous and individually linkable. Access to data is subject to prior training and authorisation and needs approval by the independent French data protection authority (“Commission Nationale Informatique et Libertés”). Data cannot be shared publicly because it is forbidden by law (sensitive individual data). Data are available from the Health Data Hub (contact via hdh@health-datahub.fr) for researchers who meet the criteria for access to confidential data.

## Abbreviations

CMUc: Complementary health universal coverage
ED: Emergency department
FDep: Social deprivation index
GPP: General practitioner or paediatrician
ICD-10: International classification of diseases 10th revision
LTD: Long-term chronic disease
SNDS: French national health data system
SSH: Short-stay hospitalisation
IQR: Interquartile range
OR: Odds ratio
95%CI: 95% Confidence interval

## References

[1] Whitfill T, Auerbach M, Scherzer DJ, Shi J, Xiang H, Stanley RM (2018). Emergency care for children in the United States: epidemiology and trends over time. J Emerg Med.

[2] Kwak YH, Kim DK, Jang HY (2012). Utilization of emergency department by children in Korea. J Korean Med Sci.

[3] Ruzangi J, Blair M, Cecil E, Greenfield G, Bottle A, Hargreaves DS (2020). Trends in healthcare use in children aged less than 15 years: a population-based cohort study in England from 2007 to 2017. BMJ Open.

[4] To T, Terebessy E, Zhu J, Fong I, Liang J, Zhang K (2021). Did emergency department visits in infants and young children increase in the last decade? An Ontario. Canada Study BMJ Paediatr Open.

[5] Riva B, Clavenna A, Cartabia M, Bortolotti A, Fortino I, Merlino L (2018). Emergency department use by paediatric patients in Lombardy Region, Italy: a population study. BMJ Paediatr Open.

[6] Boisguérin B. Urgences : Plus du quart des passages concernent les enfants de moins de 15 ans. La moitié des patients restent moins de deux heures, hormis ceux maintenus en observation Etudes et résultats. N° 1128, 2019. https://www.data.gouv.fr/fr/datasets/.

[7] Boisguérin B, Valdelièvre H. Urgences : la moitié des patients restent moins de deux heures, hormis ceux maintenus en observation. Etudes et résultats. N° 889, 2014. https://www.data.gouv.fr/fr/datasets/.

[8] Zachariasse JM, Borensztajn DM, Nieboer D, Alves CF, Greber-Platzer S, Keyzer-Dekker CMG (2020). Sex-specific differences in children attending the emergency department: prospective observational study. BMJ Open.

[9] Carlson LC, Kim J, Samuels-Kalow ME, Yun BJ, Terry DF, Weilburg JB (2021). Comparing neighbourhood-based indices of socioeconomic risk factors and potentially preventable emergency department utilization. Am J Emerg Med.

[10] Pergeline J, Rivière S, Rey S, Fresson J, Vilain A, Rachas A, Tuppin P (2023). Social deprivation level and use of healthcare services over one year by children less than 18 years of age in 2018–19: a French nationwide observational study. PLoS One..

[11] Coughlan CH, Ruzangi J, Neale FK, Nezafat Maldonado B, Blair M, Bottle A (2022). Social and ethnic group differences in healthcare use by children aged 0–14 years: a population-based cohort study in England from 2007 to 2017. Arch Dis Child.

[12] McKenzie K, Dudevich A, Costante A, Chen XK, Foebel AD (2021). How children and youth with medical complexity use hospital and emergency department care across Canada. Healthc Q.

[13] Zunino L, Colineaux H, Claudet I, Bréhin C (2021). Description of a migrant pediatric population visiting the Toulouse Children's Hospital emergency department. Arch Pediatr.

[14] Coster JE, Turner JK, Bradbury D, Cantrell A (2017). Why do people choose emergency and urgent care services? A rapid review utilizing a systematic literature search and narrative synthesis. Acad Emerg Med.

[15] McHale P, Wood S, Hughes K, Bellis MA, Demnitz U, Wyke S (2013). Who uses emergency departments inappropriately and when - a national cross-sectional study using a monitoring data system. BMC Med.

[16] Tiller R, Chan K, Knight JC, Chafe R (2021). Pediatric high users of Canadian hospitals and emergency departments. PLoS One.

[17] Supat B, Brennan JJ, Vilke GM, Ishimine P, Hsia RY, Castillo EM (2019). Characterizing pediatric high frequency users of California emergency departments. Am J Emerg Med.

[18] Vrijlandt S, Nieboer D, Zachariasse J, Oostenbrink R (2022). Characteristics of pediatric emergency department frequent visitors and their risk of a return visit: a large observational study using electronic health record data. PLoS ONE.

[19] Ravi N, Gitz KM, Burton DR, Ray KN (2021). Pediatric non-urgent emergency department visits and prior care-seeking at primary care. BMC Health Serv Res.

[20] Cecil E, Bottle A, Cowling TE, Majeed A, Wolfe I, Saxena S (2016). Primary care access, emergency department visits, and unplanned short hospitalizations in the UK. Pediatrics.

[21] Kirby S, Wooten W, Spanier AJ (2021). Pediatric primary care relationships and non-urgent emergency department use in children. Acad Pediatr.

[22] Rachas A, Gastaldi-Ménager C, Denis P, Barthélémy P, Constantinou P, Drouin J (2022). The economic burden of disease in France from the National Health Insurance Perspective: the healthcare expenditures and conditions mapping used to prepare the French social security funding act and the public health act. Med Care.

[23] Tuppin P, Rudant J, Constantinou P, Gastaldi-Ménager C, Rachas A, de Roquefeuil L (2017). Value of a national administrative database to guide public decisions: From the système national d’information interrégimes de l’Assurance Maladie (SNIIRAM) to the système national des données de santé (SNDS) in France. Rev Epidemiol Sante Publique.

[24] Décret n° 2011–77 du 19 janvier 2011 portant actualisation de la liste et des critères médicaux utilisés pour la définition des affections ouvrant droit à la suppression de la participation de l'assuré. Journal officiel de la République française. n°0017 du 21 janvier 2011.

[25] Rey G, Jougla E, Fouillet A, Hémon D (2009). Ecological association between a deprivation index and mortality in France over the period 1997–2001: variations with spatial scale, degree of urbanicity, age, gender and cause of death. BMC Public Health.

[26] Tuppin P, Drouin J, Mazza M, Weill A, Ricordeau P, Allemand H (2011). Hospitalization admission rates for low-income subjects with full health insurance coverage in France. Eur J Public Health.

[27] Wasserstein RL, Schirm AL, Lazar NA (2019). Moving to a world beyond “p < 0.05”. Am Stat.

[28] Schlichting LE, Rogers ML, Gjelsvik A, Linakis JG, Vivier PM (2017). Pediatric emergency department utilization and reliance by insurance coverage in the United States. Acad Emerg Med.

[29] Rosychuk RJ, Chen AA, McRae A, McLane P, Ospina MB, Hu KJ (2022). Age-varying effects of repeated emergency department presentations for children in Canada. J Health Serv Res Policy..

[30] Siekman N, Hilger R (2018). High users of healthcare: strategies to improve care, reduce costs. Cleve Clin J Med.

[31] Hellmann R, Feral-Pierssens AL, Michault A, Casalino E, Ricard-Hibon A, Adnet F (2021). The analysis of the geographical distribution of emergency departments' frequent users: a tool to prioritize public health policies?. BMC Public Health.

[32] Leyenaar JK, Schaefer AP, Freyleue SD (2022). Prevalence of children with medical complexity and associations with health care utilization and In-hospital mortality. JAMA Pediatr.

[33] McKenzie K, Dudevich A, Costante A, Chen XK, Foebel AD (2021). How children and youth with medical complexity use hospital and emergency department care across Canada. Healthc Q..

[34] Pergeline J, Rey S, Fresson J, Debeugny G, Rachas A, Tuppin P (2023). Factors associated with hospital admission, and 30 day readmission for children less than 18 years old in 2018 in France: a one year nationwide observational study. BMC Health Serv Res.

[35] Ismail SA, Gibbons DC, Gnani S (2013). Reducing inappropriate accident and emergency department attendances: a systematic review of primary care service interventions. Br J Gen Pract.

[36] Whittaker W, Anselmi L, Nelson P, O'Donnell C, Ross N, Rothwell K (2019). Investigation of the demand for a 7-day (extended access) primary care service: an observational study from pilot schemes in England. BMJ Open.

[37] Whittaker W, Anselmi L, Kristensen SR, Lau YS, Bailey S, Bower P (2016). Associations between extending access to primary care and emergency department visits: a difference-In-differences analysis. PLoS Med.

[38] Pergeline J, Lesuffleur T, Rey S, Fresson J, Rachas A, Tuppin P (2023). Characteristics, diseases and one year health care services utilization of children under 18 years-old in 2018–19: a French nationwide observational study. Arch Ped.

